# L1CAM is expressed in triple-negative breast cancers and is inversely correlated with Androgen receptor

**DOI:** 10.1186/1471-2407-14-958

**Published:** 2014-12-15

**Authors:** Kai Doberstein, Karin Milde-Langosch, Niko P Bretz, Uwe Schirmer, Ayelet Harari, Isabell Witzel, Alon Ben-Arie, Michael Hubalek, Elisabeth Müller-Holzner, Susanne Reinold, Alain G Zeimet, Peter Altevogt, Mina Fogel

**Affiliations:** Tumor Immunology Programme, D015, German Cancer Research Center, Heidelberg, Germany; Department of Gynecology, University Medical Center Hamburg-Eppendorf, Hamburg, Germany; Chemical Laboratories, Kaplan Medical Center, Rehovot, Israel; Department of Gynecology, Kaplan Medical Center, Rehovot, Israel; Department of Gynecology and Obstetrics, Medical University of Innsbruck, 6020 Innsbruck, Austria; Institute of Pathology, Medical University of Innsbruck, 6020 Innsbruck, Austria

**Keywords:** Triple-negative, Basal-like, ER/PR, EMT, AR

## Abstract

**Background:**

Breast cancer is a heterogeneous disease displaying distinct molecular features and clinical outcome. The molecular profile of triple-negative breast cancers (TNBCs) overlaps with that of basal-like breast cancers that in turn show similarities with high-grade serous ovarian and endometrial carcinoma. L1CAM is an established biomarker for the latter cancers and we showed before that approximately 18% of primary breast cancers are positive for L1CAM and have a bad prognosis. Here we analysed the expression of L1CAM breast cancer subtypes.

**Methods:**

We analyzed mRNA and protein expression data from different breast cancer cohorts for L1CAM, estrogen receptor, progesterone receptor, Her-2 and Androgen receptor (AR) and correlated the data. We performed Western blot analysis on tumor cell lysates and carried out chromatin-immuno-precipitation (CHIP) after AR overexpression.

**Results:**

We find that L1CAM is expressed preferentially though not exclusively in TNBCs. Using the human cancer genome atlas database and two independent breast cancer cohorts we find that L1CAM is inversely correlated with androgen receptor (AR) expression. We found that L1CAM^high^AR^low^ primary breast tumors have the worst clinical outcome. Overexpression of AR in MDA-MB436 breast cancer cells decreased L1CAM expression at the protein and mRNA level and CHIP-analysis revealed binding of AR to the L1CAM promoter region.

**Conclusions:**

These results suggest that L1CAM in breast cancer is under AR control. The data also strongly advocate the use of L1CAM assessment in breast cancer diagnosis. We suggest that L1CAM expression could be causally related to the bad prognosis of TNBCs.

**Electronic supplementary material:**

The online version of this article (doi:10.1186/1471-2407-14-958) contains supplementary material, which is available to authorized users.

## Background

Breast cancer represents a heterogeneous disease with many distinct molecular features. In clinical practice the classification according to estrogen receptor (ER), progesterone receptor (PR) or the EGF receptor family member Her-2 is prominent as these markers influence therapeutic options. Triple-negative breast cancers (TNBCs) lack all of these markers and are an aggressive subtype with high rates of proliferation and poor prognosis [1, 2]. In recent years, novel classifications based on the transcriptome analysis of breast cancer tissues have been established that revealed subtypes with distinct gene expression signatures and clinical outcome [3, 4]. Several intrinsic breast cancer subtypes were established with luminal A, Luminal B, Her-2-enriched, and basal-like (BLBC) being major groups. Recent studies have further expanded this classification [5]. The majority of TNBCs are of the BLBC subtype, i.e. often carry p53 mutations and express cytokeratin 5/6 or EGFR [6]. But these characteristics are occasionally also found in ER+ or Her-2+ breast cancers [6, 7].

Beside the hormone receptors ER and PR, more than 70% of primary breast cancers express the androgen receptor (AR) [8–10]. Recent work has shown that AR expression status is a prognostic marker in non-basal TNBCs [11] and that expression of AR is associated with better OS and DSF irrespective of co-expression of ER [10]. The loss of AR expression was also shown to predict early recurrence in TNBCs and BLBCs [12]. Recently, Santagata et al have shown that the combined analysis of AR, vitamin D receptor, ER and PR can improve the outcome prediction [13]. Other studies have used a 5–marker immunohistochemical panel comprising ER, PR, Her-2, EGFR and Ck5/6 to identify a basal-like subgroup within TNBCs [14]. Using gene expression profiling Lehmann et al. have defined 6 TNBC subtypes and identified cell lines that can serve as models for these subtypes [15]. Thus, novel markers could lead to better classification schemes associated with patient survival differences and offer novel insights for the treatment breast tumors [13].

L1CAM, a transmembrane cell adhesion molecule of the Ig superfamily, plays an important role in the development of the nervous system and in the malignancy of human tumors [16]. L1CAM is overexpressed in many human carcinomas and augments cell motility, invasion and metastasis formation [17]. Several studies have shown that L1CAM positive carcinomas have a bad prognosis [16, 18]. For gynecological cancers we reported before that type II tumors, representing the most aggressive serous and clear-cell endometrial and ovarian carcinoma, are positive for L1CAM [19–21]. In addition, a recent study has shown that the less aggressive endometrioid EC (type I tumors) can sometimes express L1CAM conferring a bad prognosis to those patients [22]. Thus, L1CAM is a novel biomarker for the prognosis of serous ovarian and endometrial cancers.

We reported before that L1CAM is also expressed in appr. 18% of primary breast cancers [23]. However, in this study we did not analyse particular subtypes for L1CAM. Given the recent findings about the molecular similarities between high-grade serous ovarian, endometrial cancers and certain forms of breast cancer [5, 24] we re-investigated L1CAM in breast cancer in more detail. In the present study we report that L1CAM is expressed in TNBCs and is inversely correlated with the expression of AR. Our results warrant the use of L1CAM staining for the improved diagnosis of primary breast cancer and suggest a link between L1CAM expression and the general bad prognosis of TNBCs.

## Methods

### Patients

The study was performed in accordance with the principles of the Helsinki declaration after approval by the local ethics committee (University of Innsbruck, Austria; University of Hamburg). Histological typing was evaluated on H&E stained sections according to the criteria of the WHO. Written informed consent was obtained from all patients for use of the resected samples. Two cohorts of primary breast cancer were analysed: 1) The Hamburg cohort of 219 patients in which the L1CAM expression was initially analysed [23] was re-investigated for AR expression. 2) A second cohort of 60 patients with invasive ductal carcinoma was recruited from Innsbruck University hospital. All were of the TNBC subtype. The clinical and histopathological data of both patient cohorts are summarized in Table 1.

**Table 1:** **Clinical and histopathological characteristics of the Hamburg and the Innsbruck cohorts of breast cancer patients**

	Hamburg n =	Innsbruck n =
Total number of cases	219	60
Patient data available	198	53
Date of surgery:	1990 - 2003	2001 - 2010
Age:		
Mean (year)	56.8	58.7
Median (year)	56.6	58.5
Range	29.3 - 93.5	33 - 84
Stage (pT)		
1	51	3
2	122	27
3	10	28
4	10	0
Unknown	5	2
Nodal status		
Negative	137	33
Positive	60	25
Unknown	1	2
Grading		
G1	20	1
G2	81	42
G3	92	15
Unknown	5	0
Histological type		
Ductal	141	60
Lobular	31	0
Others	22	0
Unknown	4	0
Estrogen receptor (ER) status^1^		
Positive	149	0
Negative	41	60
Unknown	8	0
Progesterone receptor (PR) status^1^		
Positive	125	0
Negative	65	60
Unknown	8	0
HER2 (ErbB2) status^2^		
Positive	44	0
Negative	152	60
Unknown	2	0
Molecular subtype^2^		
Luminal	121	0
HER2-positive	45	0
Triple-negative	30	60
Unknown	2	0
Adjuvant chemotherapy		
Yes	124	56
No	69	2
Unknown	5	2
Follow-up period (months)		
Mean	120.4	67.4
Median	132.0	62.9
Recurrences	72	9
Died of disease	56	8

^1^based on routine immunohistochemical ER/PR staining.

^2^based on mRNA data using the cut-off values as described [23].

### Antibodies

The mAb to the ectodomain of L1CAM (L1-14.10) was previously described [25]. Antibodies to AR (SP107, Ventana 760-4605), ER (Novocastra, NCL-L-ER-6F11, ERα specific) and PR (Zymed, PR-2C5) were used for IHC.

### Immunohistochemical staining and evaluation of expression

IHC staining was performed as described in detail recently [26]. Briefly, 3-4 μm thick histological paraffin sections were cut and mounted on Superfrost Plus slides that were exposed in a pressure cooker to EDTA pH 8.0 buffer for antigen retrieval. An automated immunohistochemistry procedure was performed using the I6000 (Biogenics, San Ramos, CA) immunostainer. Endogenous peroxidase activity was blocked by a 10 min treatment with 3% H_2_O_2_ in methanol. Slides were incubated with primary antibodies for 45 min and immunoperoxidase staining was accomplished using the Supersensitive detection kit with AEC or DAB (Zymed) as the substrates. Counterstaining was performed using hematoxilin prior to coverslipping and viewing by light microscopy. Omission of the primary antibody was used as a negative control. Positive AR expression was defined as >/=10 % nuclear staining.

### Biochemical analysis

SDS-PAGE under non-reducing conditions and transfer of proteins to an Immobilon membrane using semi-dry blotting has been described [27]. After blocking with 5% skim milk in TBS, the blots were developed with the respective primary antibody followed by peroxidase conjugated secondary antibody and ECL (Perkin Elmer, Rodgau, Germany) detection.

### Transient transfection and CHIP analysis

Transient transfection of MDA-MB 436 cells was done using jetPEI (Polyplus, Illkirch, F). 1 × 10^5^ cells were seeded 24 h before transfection in 6-well plates. The AR-GFP plasmid (Plasmid 28235: pEGFP-C1-AR) was obtained from Addgene [28]. The transfections were done as indicated in the manufacturer’s protocol and cells were selected for analysis after 48 hr. For ChIP assays, cells were seeded in 175 cm^2^ dishes, transfected either with pcDNA3 control or AR plasmid, respectively. Cells were harvested 72 h after transfection. CHIP analysis with mAb to AR was done exactly as described by the manufacturer (Active Motif). Primers for the RT-PCR analysis of AR binding sites in the L1CAM promoter were designed and synthesized by MWG Eurofines (Ebersberg, Germany). ChIP primer pair PP1: forward: ACCTTCCTCCTCCTTCTAGGC; reverse: GAGCGGTGGAAGACAGACAAA. ChIP primer pair PP2: forward: AACAAGGCTTTCCTCTGGCT; reverse: ACAGGGCACATGAAAGGGTC.

### Quantitative RT- PCR

Total RNA was isolated using the Qiagen RNeasy mini kit (Qiagen Hilden, Germany). Reverse transcription into cDNA was performed using RevertAid First Strand cDNA Synthesis Kits (Fermentas, St. Leon-Rot, Germany). For qPCR the cDNA was purified on Microspin G-50 columns (GE Healthcare, München, Germany) and quantified by NanoDrop spectrophotometer (ND-1000, Kisker-Biotechnology, Steinfurt, Germany). Primers for qPCR were designed with the DNA Star Program and were produced by MWG Eurofines (Ebersberg, Germany). ß-actin was used as an internal standard. The PCR reaction was performed with the SYBRgreen mastermix (Applied Biosystems, Darmstadt, Germany). The sequence of primers used were as following: L1CAM primer pair, forward: GACTACGAGATCCACTTGTTTAAGGA; reverse: CTCACAAAGCCGATGAACCA. Actin primer pair, forward: GGACTTCGAGCAAGAGATGG; reverse: AGCACTGTGTTGGCGTACAG. AR primer pair, forward: ACAGGAGGAAGGAGAGGCTT; reverse: ACTACACCTGGCTCAATGGC.

### FACS analysis

The staining of cells with mAbs to L1CAM and PE-conjugated secondary antibodies has been described [27]. Stained cells were analysed with a FACS Canto II using FlowJo software (Becton & Dickinson, Heidelberg, Germany).

### Statistical analysis

P-values were calculated by an unpaired t-test and 95% confidential intervals (CI) were calculated with the Graph Prism program. Survival curves were plotted by Kaplan-Meier analysis and Log-Rank tests using the SPSS.21 program. Cox regression models were calculated for multivariate analysis including classical prognostic markers. Probability values less than 0.05 were regarded as statistically significan.

## Results

### L1CAM and AR are negatively correlated in the TCGA breast cancer database

**Figure 1:**
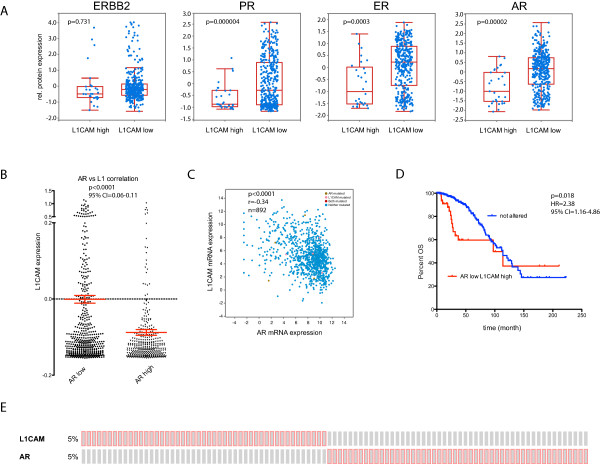
**L1CAM mRNA expression in the TCGA breast cancer collective negatively correlates to AR protein and mRNA expression.** Analysis of data from the Breast Invasive Carcinoma (TCGA, Provisional) study using cBioportal. The dataset includes 1002 cases of breast cancer patients. All correlation values were calculated by spearman. **(A)** Differences in ERBB2, PGR, AR and PGR protein expression of samples that express high amounts of L1CAM mRNA (L1CAM high: Expression > 1.3) to unaltered samples (L1CAM low: Expression < 1.3). **(B)** L1CAM mRNA expression of specimens that express low (AR low, n = 536) or high (AR high, n = 356) amounts of AR mRNA. P < 0.0001, 95% CI = 0.06-0.11. **(C)** Scatter plot of L1CAM mRNA expression to AR mRNA expression. Values are given in (RNA Seq V2 RSEM) in log2. n = 892, r = -0.34, P < 0.0001. **(D)** Kaplan Meier analysis of month survival of cases that express high amounts of L1CAM (Expression > 1.3) together with low amounts of AR (Expression < 1.3) (red line) compared to not altered patients (blue line). P = 0.018, HR = 2.38, 95% CI = 1.16-4.86. **(E)** Cases set showing overexpression of L1CAM (5%, Expression > 1.3) or AR (5%, Expression > 1.3). Red boxes represent cases with expression > 1.3; gray boxes: unaltered or < 1.3.

We analysed the TCGA collective Breast Invasive Carcinoma (TCGA, Provisional) with the cBioportal tool (http://www.cbioportal.org). The database includes 1002 cases of primary breast cancer patients and has protein and mRNA expression data. Analyzing the mRNA data for L1CAM expression, we observed high expression in 5% (n = 50) of the cases. Next, we analyzed the positive and negative groups for differential protein expression of ER, PR, ERBB2 and AR. There was an inverse correlation with protein expression of ER (P = 0.0003), PR (P = 0.000004) but not ERBB2 (P = 0.731) in agreement with our previous work [23]. Importantly, we also observed a highly significant inverse correlation with the expression of AR (P = 0.00002) (Figure 1A).

We next divided the TCGA mRNA data into AR^low^ (n = 536) or AR^high^ (n = 356) groups and carried out expression analysis. L1CAM was significantly more abundant in the AR^low^ (P < 0.0001) than in the AR^high^ group (Figure 1B,C). Kaplan-Meier curves showed a decreased survival of L1CAM positive breast cancer patients (Figure 1D). The mutual exclusivity of L1CAM and AR expression was observed in individual cases of the probe-set (Figure 1E).

### L1CAM expression occurs predominantly in TNBCs and BLBCs

**Figure 2:**
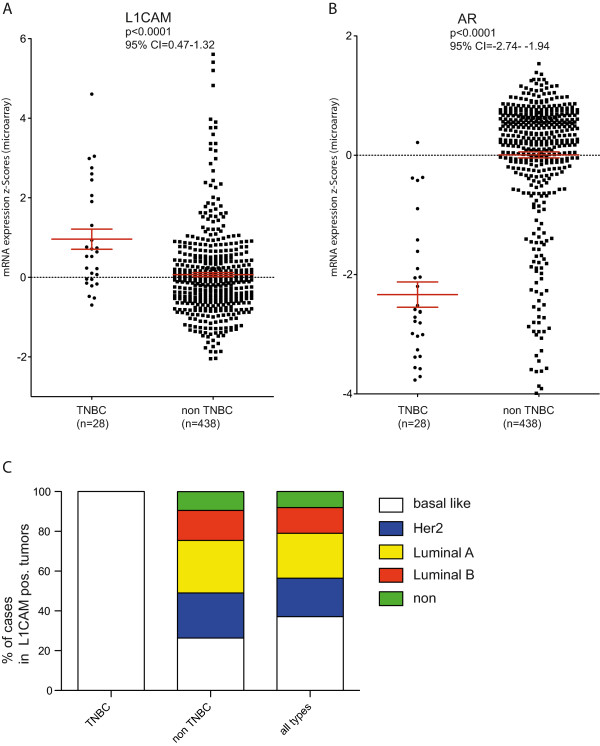
**L1CAM expression is preferentially observed in TNBCs. (A)** TNBCs (n = 28) and non-TNBCs (n = 438) from the TCGA breast cancer cohort were analyzed for the expression of L1CAM. P < 0.0001, 95% CI = 0.47-1.32 **(B)** TNBCs (n = 28) and non-TNBCs (n = 438) from the TCGA breast cancer cohort were analyzed for the expression of AR. P < 0.0001, 95% CI = -2.74- -1.94 **(C)** Distribution of histological subtypes in TNBC and non-TNBC tumors that express L1CAM.

Next the TCGA mRNA data set was stratified into TNBCs and non-TNBCs. L1CAM mRNA was increased in TNBCs versus non-TNBCs. (Figure 2A). Importantly, AR expression was decreased in TNBCs (Figure 2B) as was recently reported by others [11]. L1CAM was also enriched in BLBCs (Figure 2C).

### L1CAM and AR expression in independent breast cancer cohorts

**Figure 3:**
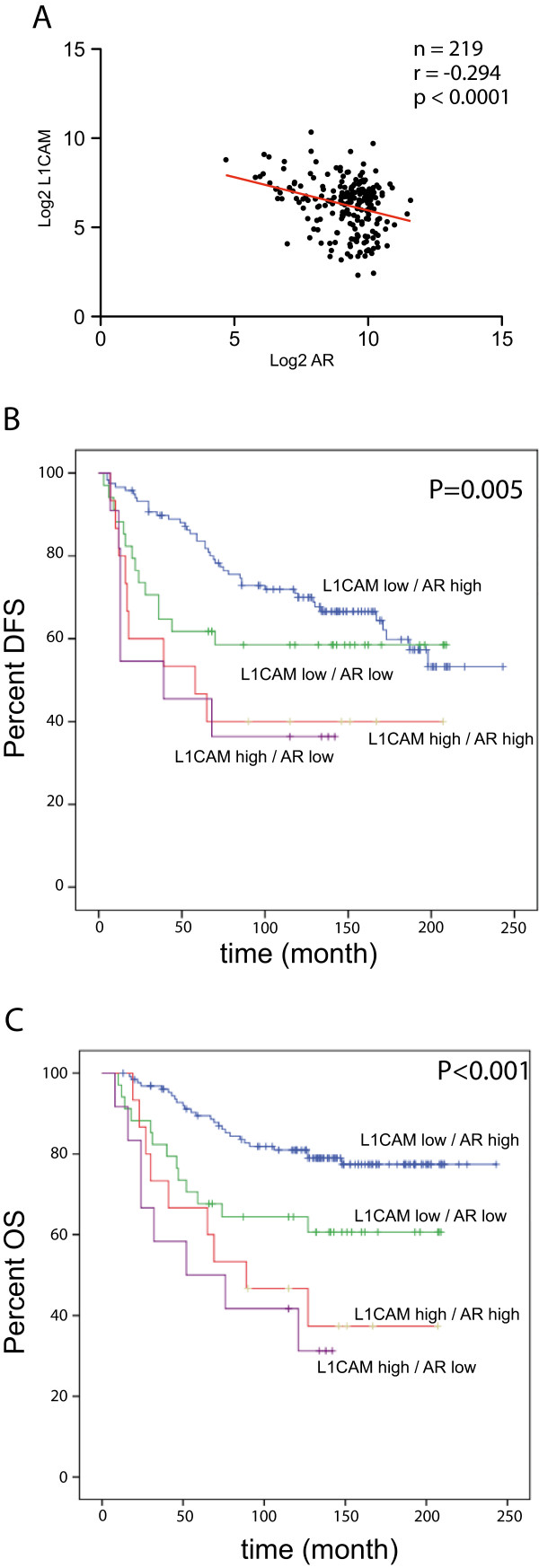
**L1CAM mRNA expression correlates negatively to AR expression in the Hamburg cohort. (A)** Scatter plot of L1CAM mRNA expression to AR mRNA expression. Values are given in (RNA Seq V2 RSEM) in log2. n = 219, r = -0.294, P < 0.0001. Kaplan Meier analysis of disease free survival **(B)** (AR pos, L1CAM neg vs. AR neg L1CAM pos: P = 0.005) and overall survival **(C)** (AR pos, L1CAM neg vs. AR neg L1CAM pos: P < 0.001) of cases that express different combination of L1CAM and AR. L1CAM^low^/AR^high^ (blue line), L1CAM^low^/AR^low^ (green line), L1CAM^high^/AR^high^ (red line), L1CAM^high^/AR ^low^ (violet line).

L1CAM was analysed before in a primary breast cancer cohort (Hamburg cohort) by mRNA expression profiling [23] (see Table 1 for clinical data). We re-analyzed the independent data set for AR expression and found a strong tendency of mutually exclusive expression between L1CAM and AR (p < 0.0001) (Figure 3A). In this cohort the inverse relation between L1CAM and ER (p = 0.043645), or PR (p = 0.020864) did reach statistical significance. Although the total numbers of TNBC and HER2-enriched tumors in the Hamburg cohort were low, the low AR expression in TNBC could be corroborated. L1CAM expression was high not only in TNBC, but also in HER2-positive tumors (Additional file 1: Table S1) that is in accordance with previous findings [23].

When the data from the Hamburg cohort were grouped according to AR and L1CAM expression, the Kaplan-Meier survival analysis revealed that L1CAM^high^AR^low^ tumors had the worst prognosis whereas L1CAM^low^AR^high^ had the best prognosis in DFS and OS (Figure 3B,C). This prognostic value retained its statistical significance in multivariate Cox regression analysis including the classical prognostic markers (FIGO stage, histological grading, lymph node involvement) showing that the L1CAM/AR status is an independent prognostic indicator for both OS and DFS (Additional file 2: Table S2).

**Figure 4:**
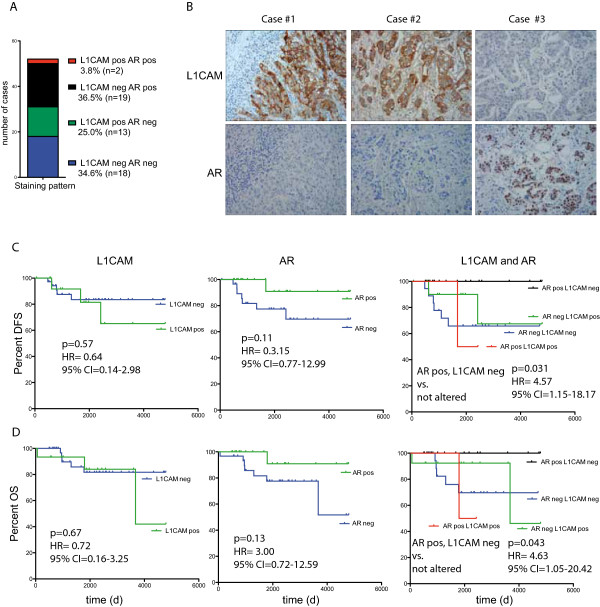
**Staining examples for L1CAM and AR breast cancer tissue sections. (A)** Staining pattern, showing the number of cases of the TNBC Innsbruck cohort that were stained by IHC for L1CAM and AR. Details are summarized in Table 1 (n = 52). **(B)** IHC staining for L1CAM and AR on representative sections from the Innsbruck cohort. Kaplan Meier analysis of disease free survival (DFS) **(C)** and overall survival (OS) **(D)** of the Innsbruck cohort that were stained by IHC for L1CAM and AR.

For validation we examined sections from a second group of patients (Innsbruck cohort). We investigated the expression of AR and L1CAM in 60 TNBCs by IHC, of which 52 cases could be evaluated for both proteins. The clinical data are summerized in Table 1. L1CAM expression was noticed in 15/52 TNBCs (Figure 4A). As expected from the results presented above, the L1CAM positive TNBCs were nearly exclusively negative for AR (13/15). But in most of the cases we detected AR specific staining in adjacent normal tissues or in L1CAM negative TNBC tumors. Examples for typical L1CAM and AR stainings are shown in Figure 4B.

When analyzing the Innsbruck cohort by Kaplan-Meier, we found that tumors that were positive for L1CAM or negative for AR had a unfavorable prognosis in DFS and OS (Figure 4C,D). These differences did not reach statistical significance most likely due to short follow-up and low sample numbers. Interestingly, the combined staining of AR and L1CAM, showed that AR positive/L1CAM negative tumors had the best prognosis for DFS (P = 0.031; HR: 4.57) and OS (P = 0.043; HR: 4.63) when compared to the rest (Figure 4C,D).

### Expression of L1CAM and AR in breast cancer cell lines

**Figure 5:**
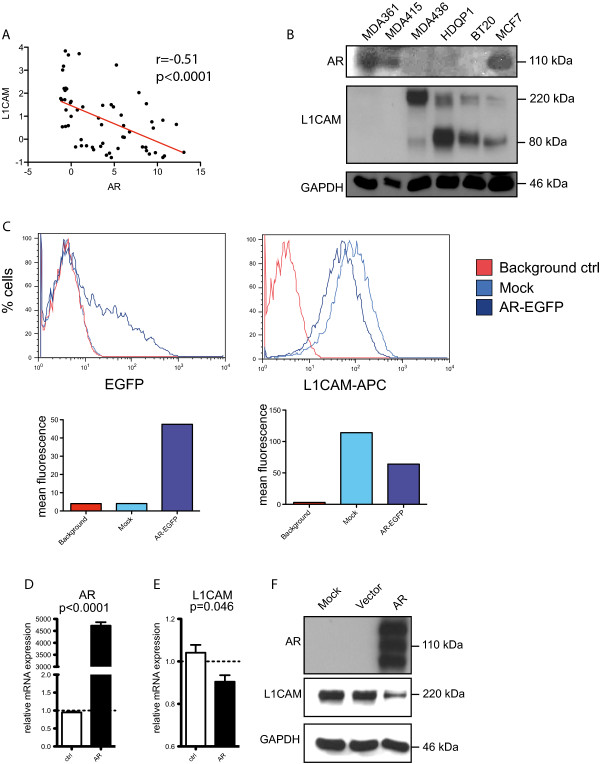
**Analysis of breast cancer cell lines for L1CAM and AR expression. (A)** The TCGA Cancer Cell Line Encyclopedia (Novartis/Broad, Nature 2012) of breast cancer cell lines was analyzed with the cBio data portal. The dataset includes 59 different breast cancer cell lines. Scatter plot showing L1CAM mRNA expression to AR mRNA expression. Values are given in mRNA Expression z-Scores. r = -0.51, P < 0.0001. **(B)** Western blot analysis of breast cancer cell lines: MDA361, MDA415, MDA436, HDQP1, BT20 and MCF7. Blot was analyzed with specific antibodies against AR, L1CAM and GAPDH as a loading control. **(C)** Representative FACS analysis of MDA436 cells that were transfected with an AR-EGFP vector or Mock. Upper row: FACS curves (% cells against log2 intensity) of EGFP and L1CAM (L1CAM-APC) stained with APC. Lower row: calculated mean fluorescence of each curve (n = 4). Transfected cells of **(C)** were analyzed by qPCR for AR **(D)** and L1CAM **(E)** expression. **(F)** Western blot analysis of MDA436 cells that were transfected with AR-EGFP, an empty vector or mock. Blots were analyzed with specific antibodies against AR, L1CAM and GAPDH as loading control (n = 3).

We next tried to confirm the inverse correlation of L1CAM and AR in in breast cancer cell lines. We analysed TCGA expression data from 59 breast cancer cell lines [5] (http://www.cbioportal.org). Dot plot analysis of L1CAM mRNA expression versus AR gave a z-score r = -0.51, (P < 0.0001) (Figure 5A). These data confirmed that AR expression is inversely correlated to L1CAM in primary tumor tissues and in breast cancer cell lines.

We selected 6 cell lines with low or high AR expression and carried out biochemical analysis. Indeed, AR^high^ cells (MDA-MB361, MDA-MB415) expressed no L1CAM as detected by Western blot analysis (Figure 5B). In contrast, AR^low^ expressing cells (MDA-MB436, HDQP1, BT20) expressed high levels of L1CAM (Figure 5B). MCF-7 cells expressed AR and low levels of L1CAM (Figure 5B).

### Overexpression of AR leads to L1CAM down-regulation

For further analysis we selected MDA-MB436 cells because of its low levels of AR expression. We transiently over-expressed AR and FACS analysis of GFP expression showed appr. 20% transfected cells after 48 hr (Figure 5C) and RT-PCR analysis confirmed strong expression of AR (Figure 5D). Importantly, L1CAM expression was decreased (Figure 5E). Staining of the transfected cells for L1CAM revealed a clear reduction at the cell surface (Figure 5C). These results were confirmed by WB analysis of total L1CAM (Figure 5F).

### CHIP analysis shows binding of AR to the L1CAM promoter

**Figure 6:**
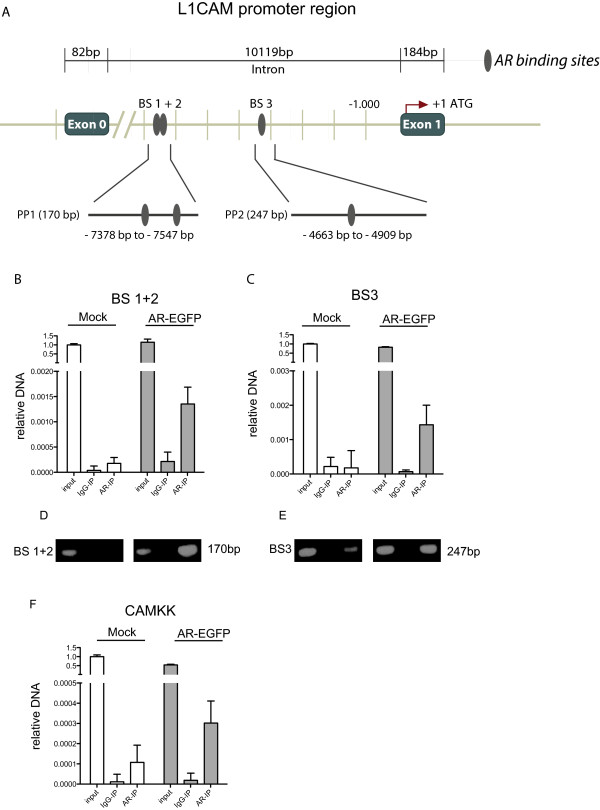
**AR binds to sites located between Exon 0 and Exon 1 of the L1CAM gene. (A)**, Schematic representation of the localization of the AR binding sites in the L1CAM gene. Upper row: Distal localization of the AR binding sites in relation to the L1CAM promoter region. Middle row: localization of binding sites 1 and 2 (BS 1+2) and binding site 3 (BS 3) between Exon 0 and Exon 1. Lower row: The localizations of primer products PP1 and PP2 are shown. An immune-precipitation (IP) of MDA436 cells transfected with AR-EGFP or mock was performed with an AR (AR-IP) antibody or a IgG (IgG-IP) control antibody. Precipitated DNA was analyzed by qPCR amplification for BS 1+2 **(B)** and BS 3 **(C)** (n = 3). The input was used as a positive control. Agarose gel electrophoresis of the BS 1+2 **(D)** and BS 3 **(E)** amplification products. **(F)** Precipitated DNA was analyzed by qPCR amplification for the AR binding site in the *CAMKK* gene. Note that the same AR-IP material was used but CAMKK specific primers (n = 3).

AR is a transcription factor and we reasoned that it might regulate L1CAM expression by binding to the L1CAM promoter. We examined the structure of the *L1CAM* promoter for putative AR binding sites. As indicated in Figure 6A, the DNA sequence analysis predicted three binding sites between exon 0 and exon 1 which is part of the L1CAM “promoter 2” region [29]. To demonstrate the binding of AR to this promoter region, we carried out Chromatin-IP analysis. MDA-MB436 cells were transfected with the AR-GFP plasmid and Chromatin-IP was carried out using anti-AR specific mAb. To analyze the binding of AR to the predicted binding sites, the precipitated chromatin-DNA was analyzed using qRT-PCR with primers specific for the selected regions. The over-expression of AR led to a strong binding to the identified binding sites BS1, 2 and BS3 as detected by RT-PCR (Figure 6B,C). Agarose gel analysis showed that both products had the expected size of 170 or 247 bp, respectively (Figure 6D,E). For specificity control we confirmed that all immuno-precipitated DNAs showed no products with off-target control primers (not shown) and no precipitates with irrelevant control IgG (Figure 6B-E). These controls suggested that AR binding sites in the *L1CAM* promoter were specifically occupied after over-expression. Finally, AR over-expression led to binding to the promoter of *CAMKK* a known target gene of AR [30] (Figure 6F).

## Discussion

L1CAM expression has been found in gynecological carcinomas such as ovarian and endometrial cancers [19–21] but was also reported to be present in a low percentage of breast carcinomas [23]. Here we have analyzed more closely the distribution of L1CAM expression in breast cancer. We found that i) L1CAM is preferentially but not exclusively expressed in TNBCs; ii) L1CAM expression is inversely correlated with the expression of AR in three independent breast cancer cohorts; iii) L1CAM and AR are inversely correlated in breast cancer cell lines and over-expression of AR down-regulates L1CAM expression in MDA-MB436 cells; iv) upon over-expression AR binds to AR response elements in the L1CAM promoter as revealed by CHIP-analysis. These findings suggest that AR can directly control L1CAM expression in breast cancer and adds a new facet to the complex regulation of L1CAM in cancer.

In previous work we and others have studied the role of L1CAM in cell motility, invasion, chemoresistance and metastasis formation (for review see [17, 18]). For MDA-MB231 breast cancer cells we showed, that the over-expression of L1CAM or its up-regulation by the EMT-inducer TGF-β augmented matrigel invasion and migration on ECM components [31]. This was associated with activation of the NFkB signalling pathway [31]. Thus, due to its ability to trigger essential cancer-related processes, L1CAM is considered as driver of tumor progression [17].

We found that L1CAM expression is more abundant in TNBCs as compared to non-TNBCs. This was observed in the TCGA cohort and was confirmed in independent patient groups. L1CAM was also found predominantly in the BLBC group consistent with the notion that TNBCs and BLBCs largely overlap. However, although preferentially expressed in these subclasses, L1CAM is occasionally also detected in non-TNBCs, especially in HER2-positive tumors (see Additional file 2: Table S2 and [23]).

Although it is well accepted that the expression of hormonal receptors such as ER and PR is associated with better outcome in women with breast cancer the role of AR has been less well investigated despite the fact that it is expressed in many breast cancers [8]. A recent systematic review and meta-analysis of 7693 patients including 19 different studies has come to the conclusion that AR expression appears to be associated with improved OS and DSF at 3 or 5 year timepoints [10]. Beside its predictive value, AR in the absence of ER expression in TNBCs may have the potential for targeted therapy using pharmacological antagonists developed for the treatment of prostate cancer [10, 32, 33].

Gaspari et al reported that AR is significantly down-regulated in TNBCs but up-regulated in Her2+, ER+, PR+ cases [11]. We find that the expression of AR is inversely correlated with L1CAM in the TCGA database and two idependent cohort. Moreover, in the panel of 56 breast cancer cell lines a similar observation was made.

As AR is a nuclear transcription factor we investigated whether the loss or down-regulation of AR might promote L1CAM expression. In line with this notion, we observed that AR over-expression in MDA-MB436 cells suppressed L1CAM at the mRNA and protein level. We identified three AR binding sites in the L1CAM promoter region localized between exon 0 and 1. Using CHIP analysis we provided suggestive evidence that AR can bind to these sites. Thus, in breast cancer AR is a negative regulator of L1CAM expression. In endometrial carcinoma we have previously identified the transcription factor Slug as a positive regulator for L1CAM [29]. In addition, in the latter tumor L1CAM is subject to epigenetic regulation via methylation of the L1CAM promoter [34] and by miR34a [35]. Thus, the present finding showing that AR can regulate L1CAM in a direct fashion adds a novel element to the complex regulation of L1CAM in cancer.

Recent TCGA studies comparing different tumor types showed many molecular commonalities between high-grade serous ovarian, serous endometrial and BLBCs suggesting a related aetiology [5, 24]. We report here that L1CAM is a prototype gene product that is up-regulated in all three tumor entities and confers a bad prognosis to the patients. It will be interesting to study whether the stratification of these cancers based on L1CAM expression will show other commonalities presently unknown.

## Conclusion

In conclusion, the present paper strongly supports the use of L1CAM in breast cancer diagnosis and suggests a link between L1CAM expression and the general bad prognosis of TNBCs.

## Electronic supplementary material

Additional file 1: Table S1: L1CAM and AR mRNA expression in different molecular subtypes of the Hamburg cohort. (XLS 48 KB)

Additional file 2: Table S2: Multivariate Cox regression analysis including FIGO stage, histological grading, nodal involvement and L1CAM/AR combination. (XLS 22 KB)

## Abbreviations

AR: Androgen receptor
BLBC: Basal-like breast cancer
DFS: Disease-free survival
EMT: Epithelial-mesenchymal transition
ER: Estrogen receptor
Her-2: Human epidermal growth factor receptor 2
IHC: Immunhistochemistry
L1CAM: L1 cell adhesion molecule
mAb: Monoclonal antibody
OS: Overall survival
PR: Progesterone receptor
RFS: Recurrence free survival
TNBC: Triple-negative breast cancer
qRT-PCR: Quantitative real-time PCR
CI: Confidence interval
HR: Hazard ratio.
